# The Role of Exploratory Conditions in Bio-Inspired Tactile Sensing of Single Topogical Features

**DOI:** 10.3390/s110807934

**Published:** 2011-08-11

**Authors:** Raphaël Candelier, Alexis Prevost, Georges Debrégeas

**Affiliations:** Laboratoire Jean Perrin, Ecole Normale Superieure, UPMC, CNRS FRE 3231, Paris 75005, France; E-Mails: raphael.candelier@ens.fr (R.C.); alexis.prevost@lps.ens.fr (A.P.)

**Keywords:** MEMS tactile sensor array, biomimetic sensor, mechanoreceptors, receptive field, friction, topological feature localization, hyperacuity, human tactile perception

## Abstract

We investigate the mechanism of tactile transduction during active exploration of finely textured surfaces using a tactile sensor mimicking the human fingertip. We focus in particular on the role of exploratory conditions in shaping the subcutaneous mechanical signals. The sensor has been designed by integrating a linear array of MEMS micro-force sensors in an elastomer layer. We measure the response of the sensors to the passage of elementary topographical features at constant velocity and normal load, such as a small hole on a flat substrate. Each sensor’s response is found to strongly depend on its relative location with respect to the substrate/skin contact zone, a result which can be quantitatively understood within the scope of a linear model of tactile transduction. The modification of the response induced by varying other parameters, such as the thickness of the elastic layer and the confining load, are also correctly captured by this model. We further demonstrate that the knowledge of these characteristic responses allows one to dynamically evaluate the position of a small hole within the contact zone, based on the micro-force sensors signals, with a spatial resolution an order of magnitude better than the intrinsic resolution of individual sensors. Consequences of these observations on robotic tactile sensing are briefly discussed.

## 1. Introduction

The human hand is an extraordinary tool which cannot be matched by any existing robotic device. It allows us both to manipulate objects with extreme precision and to extract a wealth of information such as their shape, weight, temperature and surface texture [1,2]. These capabilities owe for a large part to the cutaneous tactile sensitivity of hands that provide information regarding the forces acting on the skin. Tactile perception is mediated by specialized nerve endings (mechanoreceptors) located in the first layers of the derma. The tactile information, contained in the sequence of spikes traveling up the afferent fibers, is processed by the central nervous system to assess various physical characteristics of the probed objects such as its curvature, overall shape, temperature. Tactile perception informs about the regions of contact, the relative skin/object motion within this contact, and the direction and intensity of the interfacial forces. This information is essential to texture discrimination, grasping tasks and precise manipulation of objects. When the sense of touch is artificially canceled, even if the other sensing modalities (such as vision) are maintained, the hand becomes clumsy [3].

The performance of the human hand has driven a large effort towards the design of skin-like sensors to incorporate tactile capabilities in humanoid robots. General designs are based on a biomimetic approach which consists in mimicking the human tactile organ by reproducing one or several of its characteristics. These range from the mechanical properties and topography of natural skin to the sensitivity, frequency bandwidths, localization and density of the various mechanoreceptors. Different types of designs have been implemented up to now and a review can be found in [4]. One can find large-area thin film devices which measure one single component of the stress field (mostly the pressure) with a relatively high sensitivity but a low spatial resolution except for some very recent designs [5–8]. Such “e-skins” (electronic skins) have the advantage of being rather flexible so they can be wrapped around robotic arms for instance. One can also find systems in which one or several discrete measuring units are used as mechanoreceptors equivalent and are distributed within an elastic material. Such units range from strain gauges to piezoelectric-electric components such as PVDF films and piezo-resistive elements [9–13]. More recently, Micro-Electro-Mechanical Systems (MEMS) micro-force sensors have been increasingly used as they allow simultaneous measurements of both pressure and shear components with an enhanced sensitivity and a highly linear and hysteresis-free output [14–18]. Still, the design of large-area, highly flexible, mechanically robust and cost-effective MEMS device remains challenging.

Beyond these technological issues, efforts are still needed to establish signal processing and tactile encoding schemes that would enable one to extract the relevant information from the measured force signals. Following Dahiya *et al.* classification [4], one can distinguish two types of tasks assigned to robotic tactile sensing, referred to as “perception for action” and “action for perception”. The first case refers to situations where tactile sensing aims at providing information on the interaction between the sensor and the object, such as the existence of slippage or rolling motion, the location of the contacting area, the direction and amplitude of the contacting forces, in order to safely manipulate the object. The signal processing should therefore produce an output which does not depend crucially on the physical properties of the object. In the second situation, tactile sensing aims at assessing object properties by active exploration. In human sensing, it has long been recognized that individuals usually adopt stereotyped patterns of interaction between the hand and the object, known as “exploratory procedure”, whose characteristics depend on the type of information sought for [19–21]. Within a given exploratory procedure however, the mechanical conditions experienced by the various mechanoreceptors are expected to greatly vary. A fundamental question thus arise to how the sensing system manage, in spite of this inevitable variability of the exploratory conditions, to produce an output that only depends on the intrinsic properties of the touched object.

In both situations, the raw response of the sensors depends on many parameters, some of which being directly related to the physical properties of the object whereas others being contextual (*i.e.*, dependent on the way the object is touched). The challenge is to consistently extract some of these parameters without a complete knowledge of the others. As an illustration of this problem, let’s imagine a robotic device whose task is to lift an object with the minimal squeezing force. If one knows the friction coefficient of the skin/object interface and its weight, then it is easy to set the minimal force. If such properties are unknown, the applied force will have to be dynamically adjusted based on the detection of interfacial slip.

Here we focus on the reverse problem, *i.e.*, how to precisely extract physical characteristics of the probed surface based on the sole output of embedded micro-force sensors. For that purpose, we study, experimentally and theoretically, how the responses of subcutaneous force sensors scanned quasi-statically (low velocity) across a micro-textured surface, depend on the exploratory conditions. The paper is organized as follows. In part 2, we describe a novel bio-inspired tactile sensor designed by integrating a linear array of 10 MEMS micro-force sensors in a millimeter thick elastomer layer. The calibration of the individual sensors by point-like indentation of the skin surface as well as the experimental set-up are detailed. In part 3, the response of the sensors to perfectly smooth surfaces in both static and frictional conditions is studied. A linear model of mechanical transduction is introduced to interpret the observed stress profiles. In part 4, the sensors’ responses to the passage of elementary topographical features on a flat substrate is measured. We examine how these responses vary with (i) the sensors’ location within the substrate/skin contact zone, (ii) the skin thickness and (iii) the normal applied force. The role of these exploratory conditions is discussed within the framework of the linear model presented before. Based on these results, in part 5, a signal processing scheme is proposed which returns the location of elementary surface defects within the contact zone. We show how the use of multiple sensors increases the spatial resolution of the tactile device well beyond the intrinsic resolution of individual sensors (hyperacuity). In part 6, the impact of this study on robotic tactile sensing and future works are discussed.

## 2. Bio-Inspired Fingertip Design and Calibration

**Figure 1.**
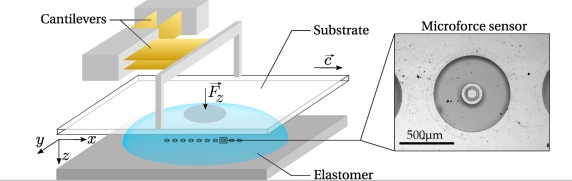
Scheme of the tactile sensing device and blow-up image of one individual micro-force sensor. (**Left**) Sketch of the experimental setup. The sensing device is made of a linear array of 10 MEMS micro-force sensors mounted on a rigid base (lower gray block), covered with a PDMS spherical cap. Flat substrates or indentor (in the form of a small rod, not shown) can be displaced and applied onto the surface of the sensing device. (**Right**) Picture of one MEMS micro-force sensor as seen from above.

The bio-inspired tactile sensor used in this study consists of a 10 mm long linear array of 10 individual MEMS micro-force sensors embedded in a spherical elastomer cap mimicking the derma-epiderma (Figure 1). The sensitive part of each micro-force sensor consists of a vertical silicon cylinder (of diameter 100 *μ*m and height 400 *μ*m) attached to a circular silicon membrane (radius 350 *μ*m, thickness 10 *μ*m) suspended on its perimeter (Figure 1, Right). The membrane bears 4 pairs of piezoresistive gauges that give access to its internal stress state, from which one can extract the force acting on the cylinder. Once covered with an elastic layer, it allows for the measurement of the three components of the subcutaneous stress in a region of lateral extension of order 200 *μ*m. Successive sensors are 1 mm apart and aligned along the *x*-axis, which is the scanning direction in dynamic sensing experiments. Sensors are numbered from left (①) to right (⑩) and a consistent color code is used in all figures, from blue to red.

The soft layer covering the sensors consists of an elastomer spherical cap with a large radius of curvature. This geometry yield an extended circular contact zone when put in contact with a flat surface. Two layer geometries were tested: a thick layer (maximum thickness *h* =3.04 mm, radius of curvature *R* =129.7 mm) and a thin layer (*h* =1.78 mm, *R* = 311.2 mm). Both were obtained by molding the liquid cross-linker/polymer melt prior to its cross-linking in a concave spherical lens whose surface has been finely abraded with a Silicon Carbide powder (The average size of abrasive grains is about 37 *μ*m. The resulting surface has a microscopic *rms* roughness of about 1.28 *μ*m whose main effect is to dramatically reduce the adhesive properties of the elastomer layer. Note that the current layer surface is not textured with fingerprint-like ridges whose effect has been discussed elsewhere [22–25]). The elastomer used here is a cross-linked PolyDiMethylSiloxane (PDMS) (The PDMS elastomer is prepared using a Sylgard 184-Dow Corning kit: dimethylvinylated and trimethylated silica are mixed with a curing agent (tetramethyltetravinylcyclotetrasiloxane) in a 10 : 1 mass ratio. Air bubbles are removed by a 5 min centrifugation at 3,000 rpm followed by exposure to a partial vacuum for a few hours. The liquid PDMS is then poured into the spherical mold, and cured at 70 °C for 48 h in an oven.) whose Young’s elastic modulus was measured to be approximately *E* =3 MPa and whose Poisson ratio was taken to be *ν* =0.5 as usually reported for this particular material.

Substrates to be scanned by the sensor, as well as the indentors used during the calibration procedure, are mounted on a three-axis motorized micro-positioning stage (micro-actuators, Newport, Inc.). A double-cantilever system combined with capacitive position sensors (MCC-30 and MCC-5, Fogale Nanotech) allows one to measure the total normal and tangential loads, respectively *F_z_* and *F_x_*, exerted on the tactile device (Figure 1, Left). In a typical experiment, the soft sensor is scanned at constant velocity *c* under a prescribed *F_z_*, across smooth or patterned Plexiglas substrates.

**Figure 2.**
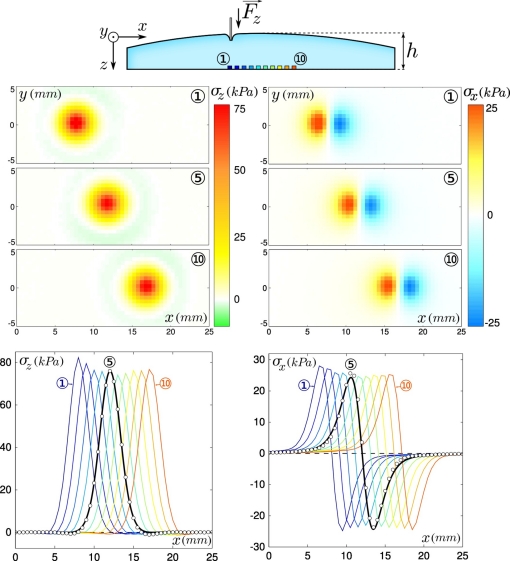
Characterization of the intrinsic receptive fields. (**Top**) Sketch showing the local indentation procedure. A 500 *μ*m in diameter cylinder is indented at the surface of the elastomer cap at varying positions (*x*, *y*). (**Middle**) Normal (left) and tangential (right) stress fields, respectively *σ_z_* and *σ_x_*, measured by the micro-force sensors ①, ⑤ and ⑩ as a function of the indentor’s position (*x*, *y*) under a unit normal force *F_z_* =1 N. (**Bottom**) Normal (left) and tangential (right) stress measured by each of the 10 micro-force sensors (from blue to red) as a function of the indentor’s position *x* for *y* =0 under a unit normal force and for a layer thickness *h* =3.04 mm. The experimental stress measured by sensor ⑤ (white circles) is compared to the prediction of a Finite Element calculation (black solid curve).

The tactile device is calibrated by applying a localized normal force at different locations on the soft layer’s surface, as depicted in the top sketch of Figure 2. A thin cylinder indentor (500 *μ*m in diameter) is iteratively loaded normally to the elastomer surface up to a prescribed load *F_z_* =0.4 N at every node of a 500 *μ*m rectangular mesh covering the entire sensitive surface of the tactile device. Regardless of the indentor’s location, the micro-force sensors response varies linearly with the applied load. By analogy to the concept of receptive fields defined in neurophysiological experiments [26], we define the Intrinsic Receptive Field (IRF) of the sensor (In contrast with the exploratory receptive fields introduced in Section 4, which depend on the exploratory conditions.) as its spatial response to a localized indentation. Typical IRF’s are shown in the middle panel of Figure 2 for both the normal and tangential stress in response to a unit load (*F_z_* =1 N) as a function of the (*x*, *y*) position of the indentor. These point-load response profiles are similar from one sensor to the next, and are centered on the sensor’s location. They are compared with the result of a Finite Elements simulation using CAST3M (CAST3M is an open source Finite Element simulation software developed at CEA, France. For additional information, see the link http://www-cast3m.cea.fr/cast3m/index.jsp. in 2*D* plane stress axisymmetric conditions. The stress field at the bottom of an elastic layer of finite thickness *h* rigidly attached to a solid plane is calculated as its surface is normally indented by a 500 *μ*m flat cylindrical indentor. In the limit where the indentor diameter is much smaller than the layer thickness, the calculated stress field is similar to that obtained for a punctual indentor (the so-called Green function) and is a function of *h* only. As shown in the bottom panels of Figure 2, the measured and calculated response profiles can be correctly adjusted for both stress components. This adjustment provides a Volt-to-Pascal calibration for each micro-force sensor, as well as an estimated value of the thickness of the layer on top of each sensor. The latter is consistent with the geometry of the elastic cap since the layer thickness is found to vary continuously along the line of sensors with a maximal thickness of the layer located close to sensor ⑥. Such thickness variation can be clearly seen on Figure 2 through an inverse variation of the maxima of *σ_z_* (corresponding to the point where the indentor lays right above the sensor).

## 3. Average Stress and Linear Model of Tactile Transduction

### 3.1. Stress Profiles Measurements

**Figure 3.**
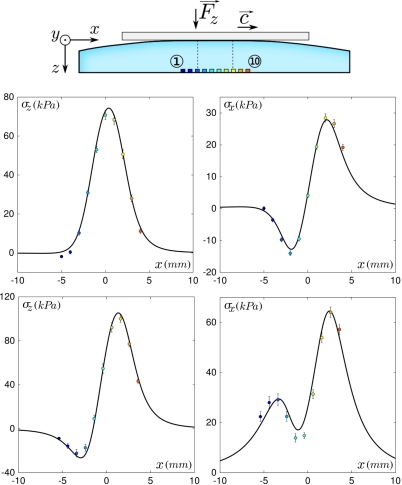
Static and dynamical mean stress as measured by the sensors and compared to the model’s predictions for a flat substrate. (**Top**) Sketch: a flat substrate is rubbed against the surface of the sensing device with a normal force *F_z_* =0.8 N(*h* =3.04 mm). Vertical dashed lines represent the boundaries of the contact zone and scale with the actual sensors’ locations. (**Middle**) Averaged stress versus sensors location *x* in the static regime (*c* =0) for the 10 sensors (colored circles) compared to the model’s prediction (black curve) for the normal component *σ_z_* (left) and similarly for the tangential component *σ_x_* along the direction of motion (right). Position *x* =0 is the center of the contact. Error bars show the measured standard deviation and colors correspond to the locations depicted on the upper sketch. (**Bottom**) Same as above but in the steady sliding regime (c = 500 *μ* m/s).

We first investigate the response of the sensing device to a smooth and flat Plexiglas substrate in both static and frictional conditions. The substrate is pressed over the device with a constant normal force *F_z_* =0.8 N, forming a circular contact area of diameter 4–5 mm (see sketch on top of Figure 3). The interfacial contact stress field is centro-symmetric with respect to the center of the contact zone, the latter being roughly above sensor ⑥. In the lower plane where sensors are located, the normal and tangential stress fields *σ_z_* and *σ_x_* (middle panels of Figure 3), are respectively symmetric and antisymmetric with respect to the center of the contact zone. A slight asymmetry in the *x*-direction is however observed. This effect can be accounted for by the development of a minute tangential force during normal loading due to the double cantilever system. In comparison, the tangential stress field *σ_y_* (not shown) is perfectly antisymmetric with respect to the *x* axis.

The substrate is then moved quasi-statically at constant velocity (*c* = 500 *μ* m/s) in the positive *x* direction (This velocity was chosen to avoid sensors breakage and skin wear. In natural physiological conditions, scanning velocities are typically in the cm/s range. However, at such velocities and for such a rigid system, the characteristic intrinsic frequencies are orders of magnitude larger than those elicited at the interface and in addition the friction coefficient for these types of materials exhibits a weak velocity dependence. One thus expects very little dynamical effects.). The average response of the micro-force sensors measured during steady sliding is shown on Figure 3, Bottom. The stress signals along the *y* axis (*i.e.*, perpendicular to the direction of motion) contain very little additional physical information as compared to *σ_x_* and *σ_z_*, and will thus be discarded from the subsequent analysis. The steady state profiles significantly differ from their static counterparts due to the development of a tangential interfacial stress field in the contact zone.

### 3.2. Linear Model of Tactile Transduction

**Figure 4.**
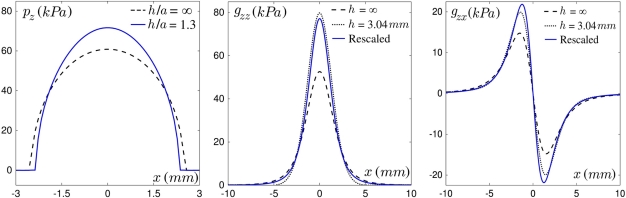
Effect of the finite thickness of the elastic layer. (**Left**) Sphere-on-plane contact pressure profile calculated for a semi-infinite elastic medium (Hertz’ calculation, dashed black line) and an elastic layer of thickness *h* (blue line) such that *h/a* =1.3 where *a* is the contact radius. This ratio corresponds to the experimental conditions. (**Center-Right**) Normal component *g_zz_* (center) and tangential component *g_xz_* (right) of the Green tensor in (*x*, *y* =0, *z* = *h*): the profile for the semi-infinite layer (dashed line) can be rescaled (blue line) to approximate the profile obtained by Finite Elements simulation for a layer of thickness *h* =3.04 mm (dotted line).

In order to interpret the subcutaneous stress profiles presented above, one first needs to calculate the stress field that develops between the elastic layer and the substrate during steady-state sliding. Unfortunately, there is no analytical solution to this mechanical problem in our confined geometry (although a solution has recently been proposed in a 2D equivalent configuration [27]). To circumvent this difficulty, we make the assumption that the normal stress field is unmodified by the application of a tangential force as would be the case in a semi-infinite configuration. Under this weakly restrictive hypotheses, an approximate semi-analytical solution can be derived whose three main ingredients are:

- *Interfacial pressure field*: The interfacial pressure field at a sphere-on-plane contact is given by Hertz’ classical calculation [28]. This result is valid in the limit of infinite semi-elastic media, *i.e.*, when the thickness of both objects is much larger than the contact radius. In our case, the latter is comparable with the elastic layer thickness. We therefore use a semi-analytical modified Hertz profile as recently proposed by Frétigny and Chateauminois [29]. As shown in Figure 4, Left, this corrected profile shows a 11% reduction in contact radius, associated with a 12% increase in maximum pressure.
- *Interfacial shear stress field*: In steady sliding regime, we postulate that at each point within the contact zone the Amontons–Coulomb Law of friction is satisfied, *i.e.*, that the local tangential stress and pressure are proportional, *p_x_* = *μ_d_*.*p_z_*, where the dynamic friction coefficient *μ_d_* ≃ 3 is evaluated as the time-averaged ratio of the total tangential to normal forces *F_x_*/*F_z_*.
- *Stress propagation in the elastic media*: Under linear elasticity and quasi-static hypothesis, the application of a force on the surface of an elastic material creates a bulk stress which is entirely set by the Green tensor **g**. The stress at point (*x*, *y*, *z*) in response to a force *f⃗* applied at the origin (*x* =0, *y* =0, *z* =0) on the surface of the material is therefore given by **g**(*x*, *y*, *z*). *f⃗*. For a semi-infinite elastic medium the analytical solution of the Green tensor is known [28] but, again, this result has to be corrected to take into account the finite thickness of the elastic layer and its attachment to a rigid base. We have carried out a finite element simulation to obtain the stress profile generated by localized normal indentation (see Section 2). This calculation indicates that a reasonable approximation of the corrected Green tensor can be obtained with a simple rescaling of the semi-infinite solution by a numerical factor k while the spatial coordinates are rescaled by k^−1/2^ to ensure force conservation. Figure 4, Center shows the normal component *g_zz_* of the Green tensor for a semi-infinite (*h* = ∞, analytical) and a finite elastic layer (*h* =3.04 mm, Finite Elements calculation). The scaling of the semi-infinite solution with a factor *k* =1.48 is also displayed for comparison. The same factor also yields a correct approximation for the tangential component of the Green tensor *g_zx_* (see Figure 4, Right). We make the assumption that corrections to the other components of the Green tensor can be obtained with the same scaling.

These ingredients being established, the stress profiles along the sensors’ line can be obtained by convoluting the interfacial stress field with the Green tensor, so that it now reads

$$\begin{array}{cc} \sigma_{\alpha }(x_{i}) & =\iint p_{x}(x,y)\cdot g_{x\alpha }(x-x_{i},y-y_{i},h)\mathrm{dxdy}\\ & +\iint p_{z}(x,y)\cdot g_{\mathrm{zx}}(x-x_{i},y-y_{i},h)\mathrm{dxdy} \end{array}$$

where (*x_i_*,*y_i_*) is the position of the sensor, and *α* ∈ {*x*, *y*, *z*}. The resulting profiles are compared to the experimental data in Figure 3. A very good agreement between the data points and the model for a value of 3.0 MPa for Young’s modulus and a Poisson ratio of 0.5, which are compatible with the material’s elastic properties, substantiates the various hypotheses and simplifications (Note that in order to account for the observed minute tangential force in the static condition, a finite tangential stress field proportional to the normal stress field has been postulated).

## 4. Response to Fine Topographical Features Scanned Across the Tactile Sensor: The Exploratory Receptive Field

### 4.1. Exploratory Receptive Fields

We now turn to the response of the tactile sensor to the passage of an elementary topographical defect on the substrate. The tactile sensor is scanned at constant velocity and confining force *F_z_* across a flat surface bearing a line of discrete holes 500 *μ*m in diameter and 1 mm in depth (The choice of using holes rather than bumps was dictated to both facilitate the fabrication of the substrates and the modelling. Upward bumps and downward holes impose different boundary conditions within the feature surface, namely a deformation for the former and a constant (zero) stress for the latter; The geometry is different from [30], since our aim here is to create a topological feature whose size is much smaller than that of the contact). Such an aspect ratio in the hole dimensions prevents the elastomer from contacting the bottom of the holes. Furthermore, the distance between neighboring holes was chosen to be larger than the contact zone diameter so that each hole produces an independent realization of the same mechanical stimulation. By scanning along adjacent parallel trajectories, one can explore the complete 2*D* response function associated with this elementary defect, for one given set of exploratory conditions.

**Figure 5.**
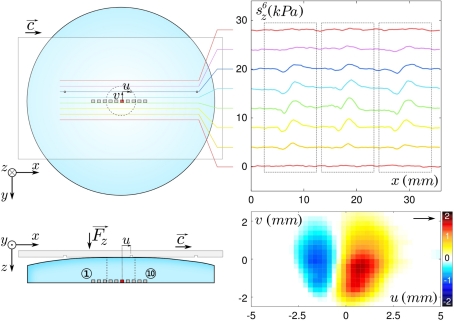
Generation of the Exploratory Receptive Fields (ERF). (**Left**) Sketches of the scanning protocol for lines of isolated defects (top: top view, bottom: side view). Dashed circle and lines represent the boundaries of the contact zone, which scale with the actual sensors’ locations. The substrate moves at constant velocity *c*. The relative position of the nearest defect to a given sensor is denoted (*u*(*t*), *v*(*t*)). **Right-Top** Normal stress responses to 3 successive defects for sensor ⑥ (*h* =3.04 mm, *F_z_* =0.8 N, *c* = 500 *μ* m/s). The raw signals, converted in stress unit, are shown for only 8 scanning lines and are shifted vertically from one to the next for clarity. **Right-Bottom** ERF of the normal stress *ς_z_* for sensor ⑥, reconstructed from the average of 14 responses to defects. Color code units are kPa.

A sketch of the experiment and the corresponding signals measured by a micro-force sensor are shown in Figure 5. Since the hole surface area is small compared to the contact zone area, its passage shows up as a small deviation from the base line (which correspond to the smooth substrate situation). These deviations remain however large with respect to the electro-mechanical noise of the sensor. We define the normal and shear responses $\varsigma_{z}^{i}$ and $\varsigma_{x}^{i}$ for each micro-force sensor *i* as the deviation of the normal and shear stress due to the presence of a defect located at position (*u*, *v*) at the interface

$$\begin{array}{l} \varsigma_{z}^{i}(u,v)=s_{z}^{i}(u,v)-\sigma_{z}^{i}\\ \varsigma_{x}^{i}(u,v)=s_{x}^{i}(u,v)-\sigma_{x}^{i} \end{array}$$

where $s_{z}^{i}(u,v)$ and $s_{x}^{i}(u,v)$ are the stress signals and $\sigma_{z}^{i}$ and $\sigma_{x}^{i}$ are the time-averaged stress already characterized in the previous section. Both *ς_z_* and *ς_x_* can be seen as an “exploratory receptive field” (ERF), which are the dynamic counterparts of the IRF displayed in Figure 2, Bottom. A typical two-dimensional ERF for the normal stress for one given micro-force sensor, constructed from an average over 14 realizations, is shown in the bottom-right panel of Figure 5.

The spatial characteristics of the ERF can be quantitatively interpreted within the scope of the linear model of tactile transduction presented in the previous section. To a first approximation, one assumes that the presence of a small hole on the flat substrate locally cancels the normal and shear stress without modifying the Hertz-like stress field in the rest of the contact zone. Under such an assumption, the defect-induced stress variations measured by the micro-force sensors can be calculated analytically. As long as the defect’s surface remains small with respect to the contact zone area, the stress variations for a sensor *i* located at (*x_i_*, *y_i_* =0) writes:

(1) $$\begin{array}{l} \varsigma_{x}^{i}(u,v)=-[p_{x}(u,v)g_{\mathrm{xx}}(u-x_{i},v-y_{i},h)+p_{z}(u,v)g_{\mathrm{zx}}(u-x_{i},v-y_{i},h)]A_{d}\\ \varsigma_{z}^{i}(u,v)=-[p_{x}(u,v)g_{\mathrm{xz}}(u-x_{i},v-y_{i},h)+p_{z}(u,v)g_{\mathrm{zz}}(u-x_{i},v-y_{i},h)]A_{d} \end{array}$$

where *A_d_* is the surface area of the defect. This calculation can be performed for any exploratory conditions and geometrical characteristics of the layer, which allows for a direct comparison with the measured ERF’s.

### 4.2. The Role of Exploratory Conditions

As outlined in the introduction, the characteristics of tactile transduction depend not only on the intrinsic properties of the tactile sensor, but also on the mechanical conditions of the frictional interaction between the substrate and the sensing device. These conditions are controlled by several quantities: some of them are contextual (namely the global applied force and the skin/substrate contact location), some are intrinsic to the sensor (the skin radius of curvature and elastic moduli), and one is characteristic of the skin/substrate interfacial interaction (the friction coefficient). In this section, we systematically vary those different parameters and analyze how they affect the measured and calculated ERF’s.

**Figure 6.**
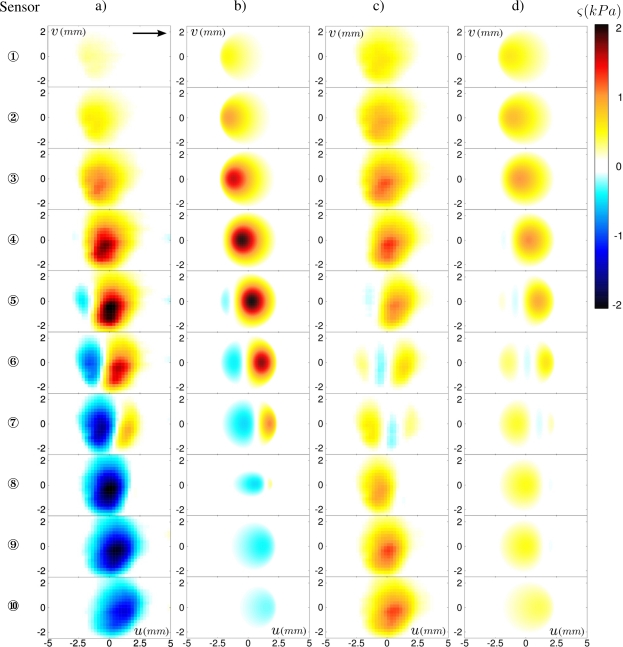
Measured and predicted ERF. Each row corresponds to one sensor, from ① to ⑩ starting from the top. Each column corresponds to the measured (**a**) and predicted (**b**) normal stress *ς_z_*, and measured (**c**) and predicted (**d**) shear stress *ς_x_*. Standard deviations are typically 0.6 k*Pa* for *σ_z_* and 0.4 *kP a* for *σ_x_*. The arrow indicates the defect’s displacement. The color code is the same for all plots. *h* =3.04 *mm* and *F_z_* =0.8 N.

The first parameter we focus on is the position of the contact zone relative to the micro-force sensors location. Figure 6(a,c) shows the two-dimensional ERF’s for all 10 sensors for both stress components under a confining force *F_z_* = 0.8 N. It appears that the passage of the small topographical feature produces a signature whose shape strongly depends on the precise location of the sensor. It is remarkable for instance that the normal ERF *ς_z_*(*u*, *v*) evolves from a positive spot for the most left-handed probing positions to a negative spot for the most right-handed ones. Note that the outmost sensors do respond to the defect’s passage although they lay outside of the contact zone.

The corresponding calculated profiles, displayed in Figure 6(b,d), correctly capture the spatial distribution experimentally observed for the ten sensors, as well as their amplitudes. There are however some discrepancies, in particular for the right-hand side sensors (from ⑥ to ⑩) whose amplitudes are systematically higher than expected both for the normal and shear stress. This may come from a coupling between tangential and normal stresses, ignored in our model: the friction-induced shear stress field produces a torque on the elastic layer which is balanced by a desymmetrization of the interfacial pressure profile (see [27] for a discussion of this effect in a cylinder/plane geometry).

This simple model captures the essential features of the position-dependence of the sensors’ response. It allows one in turn to interpret the origin of the observed variability in the ERF’s. Equation (1) shows that the signal measured by the sensor depends on the product of its intrinsic response function g by the time-averaged interfacial stress field (*p_z_*, *p_x_*). In the sphere/plane geometry used in the experiments, the latter is given by Hertz calculation (Figure 4) which rapidly varies over the contact zone. As a result, the average stress field *seen* by a given sensor, and thus its ERF, is extremely sensitive to its exact location below the contact zone.

**Figure 7.**
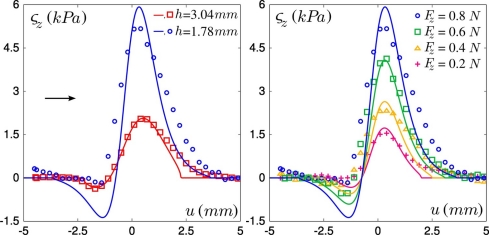
Role of two exploratory parameters: experimental data (points) at the ERF median line (*v* =0) are compared to the model prediction (solid curves) for a sensor at the center of the contact. (**Left**) Average normal stress variations *σ*_z_ for two layer thicknesses: *h* =1.78 mm (blue circles) and *h* =3.04 mm (red squares). The arrow indicates the defect’s displacement. *F_z_* =0.8 N. (**Right**) Average normal stress variations *σ*_z_ for four normal forces from 0.2 to 0.8 N (*h* =1.78 mm).

The use of two tactile devices with different layer thicknesses allows us to analyze the role of this geometrical parameter: in Figure 7, Left the recorded normal stress variations as the defect is scanned along the sensor mid-line are shown for one micro-force sensor located below the center of the contact zone, for both layers. With the thinner one, the response amplitudes are higher although the global shape of the responses is maintained. Increasing the normal applied force *F_z_* from 0.2 to 0.8 N (see Figure 7, Right) similarly increases the stress amplitude, but also widens the profiles as the contact radius *a* is increased. Notice however that this parameter weakly modifies the global shape of the responses over our experimental range.

As can be seen on Figure 7, the dependence on the layer thickness and on the normal applied force are correctly captured by our model. It should be noticed however that the quality of the fit deteriorates when the thinner layer is used, e.g., in Figure 7, Left the positive peak is broadened and the amplitude of the negative peak is smaller than predicted. This may be accounted for by the narrower intrinsic receptive fields (IRF) of the thin-layer sensor, which results in a greater sensibility to short-scale details of the surface stress fields. Discrepancies between observed and calculated profiles may thus result from the crude approximation made when estimating the local modification of the interfacial stress field induced by the hole.

**Figure 8.**
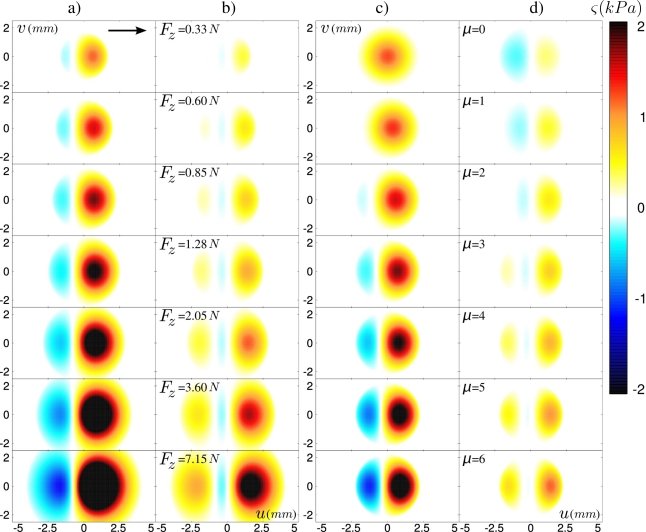
Evolution of the predicted ERF for a sensor at the center of the contact zone (*x* =0, *y* =0) in response to a single defect in (*u*, *v*) scanned at constant speed from left to right (*h* =3.04 mm, *c* = 500 *μ* m/s). Two parameters are explored: (**a–b**) the applied normal force *F_z_* and (**c–d**) the dynamic friction coefficient *μ_d_*. Columns (a) and (c) show the normal stress profiles *σ_z_*, columns (b) and (d) show the shear stress profiles in the direction of motion *σ_x_*.

Altogether, these observations validate our model of tactile transduction. The latter can further be used to overcome some experimental limitations of our tactile device: the accessible range of normal load can be extended beyond the breakage limit of the micro-force sensors, and the dynamical friction coefficient can be varied. In Figure 8(a,b), the predicted ERF for both stress components are shown for a normal force 0.33 *N* < *F_z_* < 7.15 *N*, a friction coefficient *μ_d_* = 3 and a layer thickness of *h* =3.04 mm. The ERF’s are calculated for a sensor located at the center of the contact. This series of graphs corresponds to increasing values of the contact radius *a* from 0.75 h to 1.8 h. It appears that the increase of the applied force up to large values essentially maintains the shape of the response profile, except for a scaling factor given by the ratio *a/h*. It should be noted however that the modification of the ERF is more pronounced for sensors located at the edge of the contact (not shown). For those sensors, the main effect of increasing the contact area is to move the relative position of the sensor with respect to the contact zone. Figure 8(c,d) show how varying the dynamical friction coefficient *μ_d_* in the 0–6 range modify the ERF of a centrally located sensor. In contrast to the normal applied force, an increase of the friction coefficient strongly affects the shape of the sensors’ response.

### 4.3. Discussion

In our device, the sensor base is much more rigid than its soft covering layer. This mechanical contrast complicates the calculation of both the IRF and the contact stress field. As a result, no exact scaling can be exhibited between the ERF and the various exploratory or mechanical parameters. It is therefore interesting to examine the dependence of the predicted ERF in the limit where the base and layer would have similar mechanical properties. In this case, one can show that the ERF can be written as:

$$\varsigma_{z}^{i}(\tilde{u},\tilde{v})\approx -{\tilde{A}}_{d}{\tilde{F}}^{1/3}\sqrt{1-({\tilde{u}}^{2}+{\tilde{v}}^{2})/{\tilde{F}}^{2/3}}[{\tilde{g}}_{\mathrm{zz}}(\tilde{u}-{\tilde{x}}_{i},\tilde{v}-{\tilde{y}}_{i},1)+\mu {\tilde{g}}_{\mathrm{xz}}(\tilde{u}-{\tilde{x}}_{i},\tilde{v}-{\tilde{y}}_{i},1)]$$

where all lengths are expressed in unit of *h*, $\tilde{F}=\frac{F_{z}(1-v^{2})}{ER^{2}}$ is the reduced confining force (*E*: Young’s modulus, *ν*: Poisson ratio, *R*: radius of curvature), and *g̃_zz_*, *g̃_xz_* are normalized Green functions.

This expression shows that, in this ideal situation, the ERF only depends on 3 independent parameters characterizing the exploratory conditions: *F̃*, *μ* and (*x̃_i_*, *ỹ_i_*). Notice that the amplitude of the ERF increases non-linearly with the normal load and that this parameter also controls the width of the ERF. In practice, these three parameters can be extracted from the average stress profiles, like those presented in Figure 3.

The present model has been developed to account for finite-thickness elastic layers on top of a rigid plane as generally found for most robotic tactile sensors. In human skin, however, the receptors are embedded in soft tissues which can be reasonably described by a semi-infinite elastic medium. As shown in Figure 4, the finite-size effect of the elastic layer changes only marginally both the amplitude and lateral extension of the Hertz profiles and Green functions, suggesting that the results presented here should be of relevance also for e-skin devices and biological systems.

Moreover, such an approach opens possibilities of anticipating the ERF for a given set of exploratory parameters using either the model described above or machine learning techniques. Detection of fine textural features, such as individual topological defects, thus becomes conceivable by direct comparison with the measured signals.

## 5. Predicting the Defect’s Position

The Exploratory Receptive Fields (ERF) exhibited in the previous sections are characteristic responses of the sensors, for one given set of exploratory conditions, to the passage of an elementary surface defect. Under linear hypothesis, these fields entirely characterize the transduction of textural information from the substrate to the sensors array in this particular context of exploration. In addition of being useful building blocks for the understanding of more complex tactile inputs, the complete characterization of the ERFs for isolated defects is relevant for several practical situations, like Braille reading for instance. One may then ask how much information can be retrieved by inverting the problem, *i.e.*, how precisely the position of the defect within the contact can be determined based on the sole signals delivered by the micro-force sensors.

**Figure 9.**
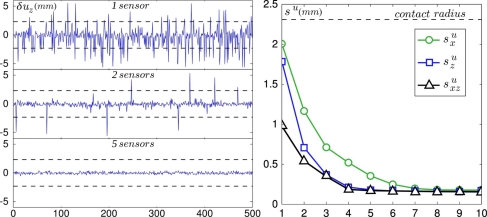
Detection of the defect’s position along the direction of motion. (**Left**) Distance between the actual and predicted positions of the defect *δu_z_* for 500 random positions of the defect in the contact zone. The predicted positions are derived by comparing the measured normal stress signals to the average stress profiles for 1, 2 and 5 sensors chosen at random for each realization. The dashed horizontal lines on all three plots are boundaries of the contact zone. (**Right**) Prediction accuracy *s^u^* as a function of the number of sensors used when only the shear stress signal is used ( $s_{x}^{u}$, green circles), then when only the normal stress is used ( $s_{z}^{u}$, blue squares) and finally when both signals are used ( $s_{\mathrm{xz}}^{u}$, black triangles). In all three cases, the prediction accuracy converges asymptotically to 150 *μ*m.

For simplicity, we focus on the prediction of the position *u* in the *x*-direction (which is both the axis of the linear array and the scanning direction) of defects scanned right above the sensors line. Our inversion procedure is based on the prior knowledge of the ERF: the averaged sensors’ response to the passage of 14 holes yields characteristic subcutaneous stress variation profiles *ς̃^i^* (*u*) for any position *u* of the hole. From this, we can now estimate the position of the hole for a given realization by evaluating the value of *u* that minimizes the following quantity

$$\xi (u)=\sum_{i\in 𝒮}{\left(\varsigma^{i}-{\bar{\varsigma }}^{i}(u)\right)}^{2}$$

where *ς^i^* is the instantaneous measured stress variation field and the sum is performed over a subset of sensors 𝒮. We are interested in both the maximum resolution thus attainable and on its dependence with the number of sensors and force components used in this estimation. First, the minimization is computed using the normal component *σ_z_* only. The difference between the predicted and the actual position of the defect, noted *δu_z_*, is shown in Figure 9, Left for 500 independent realizations, using 1, 2 and 5 sensors respectively. For each realization, regardless of the actual defect position within the contact, the subset of sensors used for the estimation is chosen randomly among all 10 available sensors. As expected, the prediction accuracy rapidly increases as the number of sensors used in the estimation, and thus the available information, increases. A similar analysis was conducted using only the *x* component of the micro-force sensors and using both *x* and *z* components. The results are summarized in Figure 9, Right which displays the evolution of the standard deviation of *δu_z_*, denoted $s_{z}^{u}$, as a function of the number of sensors used.

This graph calls for several comments. First, the spatial resolution obtained with a single sensor is of order 1–2 mm, which corresponds to the width of the IRF, *i.e.*, the intrinsic spatial resolution of individual sensors directly related to the thickness of the overlying elastomer layer. Second, the resolution length rapidly decays as the number of sensors (or equivalently the number of components) used in the inversion procedure is increased and asymptotically reaches a minimum of order 150 *μ*m when the total number of components reaches 5. Note that this value is an order of magnitude smaller than the intrinsic resolution length of each sensor, a situation classically referred to as *hyperacuity*. Finally, the normal component appears to be more efficient than the shear component in this inversion procedure as indicated by the fact that the resolution obtained using the normal component alone is systematically better than the one obtained using the shear component.

## 6. Conclusions

The work presented in this paper aimed at clarifying how the response of a tactile sensor scanned across a textured surface depends on the characteristics of the exploration procedure. In a typical bio-inspired tactile device, the responses of the embedded sensors to a localized indentation on the surface are similar from one to the next. These so-called IRFs are entirely controlled by the elastic layer’s thickness. However, the sensors’ response to the passage of a textural feature appears to strongly depend on their exact location within the skin/surface contact zone. This effect was evidenced by measuring the Exploratory Receptive Field of each sensor defined as its spatial response to the passage of a unique small feature (a circular hole) on the surface. A linear model of tactile transduction allowed us to correctly predict the form of these ERF based on the knowledge of the sensors’ IRF as well as on a few contextual parameters, namely the location of the sensor with respect to the contact zone, the applied normal load and the dynamic friction coefficient. This model allowed us to discuss the effect of each of these parameters in shaping the ERF.

This result has important consequences for robotic tactile sensing. In order to consistently retrieve textural information from the stress signals measured with an embedded sensor, one needs to know how to relate the topographical properties of the scanned surface with the measured signals. The present work indicates that this operation cannot be implemented based on the sole knowledge of the intrinsic properties of the sensor. In a structured environment, *i.e.*, when the exploratory conditions are perfectly controlled and stable, one may obtain these responses empirically by measuring the sensors’ response to isolated defects. This approach, used in Section 5, allowed us to retrieve the instantaneous position of a small defect with a much higher resolution than the intrinsic resolution of the individual micro-force sensors. In a non-controlled environment however, a different approach is needed. In this context, the linear model detailed in this paper could be useful since it allows one to predict the form of the ERF based on a few contextual parameters. The latter could be obtained dynamically by analyzing the low frequency force signals whose spatial dependence are characteristics of the averaged surface stress field (see Figure 3).

The dependence of the sensors’ response to exploratory conditions obviously complicates the inversion task. However, one may argue that it could ultimately be beneficial to the efficiency of the tactile system. For a given set of exploratory conditions, each sensor is associated with a specific spatial response and is thus sensitive to a particular type of textural features. By actively varying the exploratory procedures, one may actually tune these response functions in order to extract more detailed information on the surface. This strategy may actually exist in human tactile sensing where the exploratory procedures are known to be function of the type of information one seeks to obtain and on the physical characteristics of the probed object [19–21].

This study was limited to the response to small isolated defects. It has relevance for comparisons with neurophysiological measurements for which the ERF have been measured via reverse correlation techniques [31–33]. In more complex situations, typically when several defects lay within the contact zone, elastic interactions between the defects should be taken into account. This work should therefore be considered as a first step towards an analytical understanding of interfacial transduction of texture information.
